# Boy with a sore throat

**DOI:** 10.1016/j.acepjo.2025.100322

**Published:** 2026-01-09

**Authors:** Kenechukwu Isi, Wesley Eilbert

**Affiliations:** Department of Emergency Medicine, University of Illinois, College of Medicine, Room 469 COME, 1819 W Polk St, Chicago, IL 60612

**Keywords:** uvulitis, pharyngitis, epiglottitis

## 1 Patient Presentation

An 8-year-old boy presented to the emergency department complaining of a 1-day sore throat with difficulty swallowing due to the pain. He denied being short of breath, and there was no history of fever. On examination, he was afebrile and nontoxic in appearance. His oropharynx was erythematous with an inflamed, erythematous uvula (Fig). Swabs for streptococcal antigen and viral pathogens were negative. A soft tissue x-ray of the neck showed no evidence of epiglottitis.

**Figure:**
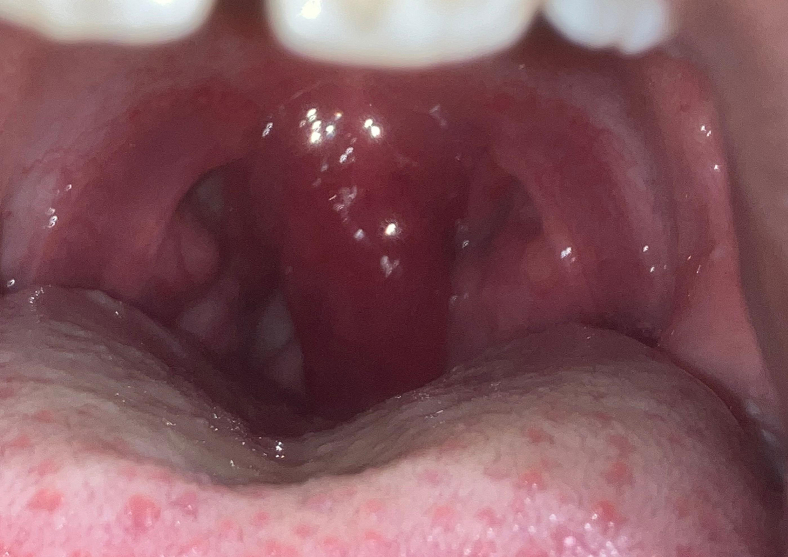
An inflamed, erythematous uvula with erythema of the oropharynx.

### 2 Diagnosis

#### 2.1 Uvulitis

Uvular edema can be caused by trauma, chemical burn, angioedema of immunologic or nonimmunologic origin, neoplasm, and viral or bacterial infection.^1^^,^^2^ Uvulitis secondary to infection is usually also associated with throat pain, odynophagia, fever, and an inflamed appearance of the uvula.^3^ Uvulitis in children has traditionally been viewed as having greater potential for infectious morbidity and may be associated with epiglottitis.^4^^,^^5^ In the era of *Haemophilus influenzae* type b vaccination, group A *Streptococcus* is now the main pathogen in cases caused by bacterial infection.^6^ Treatment of uvulitis should be tailored to the suspected cause and may include antibiotics, antihistamines, corticosteroids, nebulized racemic epinephrine, and uvular decompression.^1^^,^^7^ Admission for airway observation is generally warranted, and many authors recommend a lateral soft tissue neck x-ray or laryngoscopy to assess for concomitant epiglottitis.^1^^,^^3^^,^^5^^,^^8^^,^^9^

This patient was admitted and treated with intravenous clindamycin and dexamethasone. His symptoms improved overnight, and he was discharged the next day to complete a 10-day course of clindamycin.

## Funding and Support

By *JACEP Open* policy, all authors are required to disclose any and all commercial, financial, and other relationships in any way related to the subject of this article as per ICMJE conflict of interest guidelines (see www.icmje.org). The authors have stated that no such relationships exist.

## Conflict of Interest

All authors have affirmed they have no conflicts of interest to declare.
